# Lack of Association between Human Plasma Oxytocin and Interpersonal Trust in a Prisoner’s Dilemma Paradigm

**DOI:** 10.1371/journal.pone.0116172

**Published:** 2014-12-30

**Authors:** James C. Christensen, Pavel A. Shiyanov, Justin R. Estepp, John J. Schlager

**Affiliations:** 1 Air Force Research Laboratory, Wright-Patterson Air Force Base, Ohio, United States of America; 2 Henry M. Jackson Foundation for the Advancement of Military Medicine, Inc., Bethesda, Maryland, United States of America; University of Chicago, United States of America

## Abstract

Expanding interest in oxytocin, particularly the role of endogenous oxytocin in human social behavior, has created a pressing need for replication of results and verification of assay methods. In this study, we sought to replicate and extend previous results correlating plasma oxytocin with trust and trustworthy behavior. As a necessary first step, the two most commonly used commercial assays were compared in human plasma via the addition of a known quantity of exogenous oxytocin, with and without sample extraction. Plasma sample extraction was found to be critical in obtaining repeatable concentrations of oxytocin. In the subsequent trust experiment, twelve samples in duplicate, from each of 82 participants, were collected over approximately six hours during the performance of a Prisoner’s Dilemma task paradigm that stressed human interpersonal trust. We found no significant relationship between plasma oxytocin concentrations and trusting or trustworthy behavior. In light of these findings, previous published work that used oxytocin immunoassays without sample extraction should be reexamined and future research exploring links between endogenous human oxytocin and trust or social behavior should proceed with careful consideration of methods and appropriate biofluids for analysis.

## Introduction

Interest in the function and effects of the neuropeptide oxytocin in humans has expanded rapidly. Diverging greatly from oxytocin’s well known roles in parturition and lactation, widely-publicized results have implicated oxytocin in a range of complex social and psychological phenomena [1]. Oxytocin has been reported to induce anxiolytic effects [2], to reduce food intake and adiposity [3], to facilitate social interaction, bonding, and trust [4]–[6], to ameliorate social or emotional deficits associated with psychopathologies [7], [8], and to attenuate the effects of psychostimulants [9]. These results have significantly impacted their respective fields and have motivated ongoing research into oxytocin as a pharmacologic intervention to ameliorate causes or symptoms of various disorders [10].

The evidence supporting intervention studies and a significant role for oxytocin in psychological phenomena is generally derived from three primary sources: (1) assays of peripheral and/or central oxytocin in rodent models, (2) assays of endogenous peripheral oxytocin in humans, and (3) the results of exogenous oxytocin administration studies in humans, either via peripheral infusion or intranasal spray. The work described here focused on quantitative analysis of endogenous oxytocin in human blood plasma as a function of trusting or trustworthy behavior. While administration studies are not without concern and criticism [11], we have found that there has been limited verification of oxytocin assays leading to concerns that imprecise assays may have impacted the validity of results that have been reported in the literature to date [12]. The present study therefore focused on coupling assay verification with replication of the widely-reported link between oxytocin and trusting and trustworthy behavior.

Oxytocin is a nonapeptide produced both in the central nervous system (CNS) and many tissues throughout the periphery. The psychological effects of oxytocin are likely associated with hypothalamic production and release, but the gastrointestinal tract, heart, uterus, testes, corpus luteum, and placenta also secrete oxytocin [13]. Receptors are found in a similar variety of tissues. As will be discussed further below, there is considerable disagreement regarding typical concentrations of oxytocin in human plasma. Nevertheless, it appears that normal human circulating concentrations are well below 1 ng/mL [14]. The low concentration of oxytocin in the periphery coupled with practical considerations of cost and throughput have favored detection using immunoassays with either radiolabeling or enzyme-linked optical absorptive reporters. Such immunoassays can be highly specific and sensitive; however, this specificity and sensitivity depends upon optimized procedures as well as components including the choice of antibody and biofluid isolated for assay. The chosen biofluid (e.g., blood plasma) may contain elements that cause interference and nonspecific binding for the analyte targeted by the assay [14].

One might assume that the psychoactive properties of oxytocin are solely dependent on activity in the CNS, and consequently researchers should focus on assaying oxytocin in cerebrospinal fluid (CSF). However, there is evidence for complex feedback both within the periphery and between the CNS and the periphery [3], [11] that can result in coordinated, though not coupled, release [15]. Oxytocin also does appear to cross the blood-brain barrier, though in very small quantities [16]; thus, it is possible that peripherally released oxytocin exerts some direct influence on the CNS. As a practical matter, research on oxytocin as a biomarker in humans has most frequently involved assays of plasma levels due to the invasive nature, procedural complexity, and safety concerns associated with sampling CSF.

Research conducted with animal models has enabled the assessment of CSF oxytocin with findings that are generally consistent with the roles of oxytocin mentioned above. Some caution is warranted in extending results to human behavior, in particular from data using rodent models. Zhang et al. [17] used two-dimensional liquid chromatography-tandem mass spectrometry (2D LC-MS/MS) to demonstrate that endogenous plasma oxytocin concentrations in rats are approximately 2000-fold higher than those observed in humans. Prairie voles, another common model for oxytocin research, have been reported to exhibit endogenous oxytocin concentrations well in excess of those of rats [18]. This large mean difference in circulating levels of oxytocin is not absolute evidence of variance in physiological mechanism or effects since, for example, aspects of receptor affinity and density could be lower in rodents, thus offsetting the difference in baseline concentration. Nonetheless, there is evidence that oxytocin blood plasma levels vary significantly across mammalian models and substantial evidence that the behaviors affected by oxytocin are highly species-specific [19], [20].

There is considerable disagreement regarding typical levels for oxytocin concentration in human plasma. Restricting our search to baseline measurements of oxytocin (excluding populations that were pathological, pregnant, lactating, etc.), we identified 47 publications (Table S1 in S1 File). Undoubtedly, more exist, but this sample was sufficient to demonstrate high variability in “normal” and expected oxytocin concentrations. Average concentrations within each publication ranged from 0.5 pg/mL to 3.6 ng/mL, with a mean of 169 pg/mL across all 47 studies. In analyzing the methods used in these publications, the largest apparent contributor to this variability, by far, was the use of pre-assay sample extraction. Without any sort of extraction, 23 publications produced a mean concentration of 360.9 pg/mL (SD: 731.6), while extracted samples produced a mean of 10.4 pg/mL (SD: 20.4) in the remaining 24 publications.

Neural correlates of the Prisoner’s Dilemma paradigm have been previously explored [21], [22] and appear to involve conflict resolution and reinforcement learning over multiple rounds of the task. For the purposes of the present study, the iterated Prisoner’s Dilemma scenario meets the basic definition of trust [23]: cooperation in the task entails making oneself vulnerable to another’s actions where potential losses are greater than potential gains. Further evidence supporting the choice of paradigm was provided by a study of exogenous oxytocin administration during a Prisoner’s Dilemma study [24] which found that administration promoted in-group cooperation and defensive aggression towards competing outgroups. Consequently, we expected to observe behavior and associated plasma oxytocin levels to vary significantly with both trust/trustworthiness and the ingroup or familiarity status [5] of a task partner. While this task is different in structure than the typical trust game, as implemented by Zak, Kurzban, and Matzner [25], the common features of monetary vulnerability and outcomes contingent on joint decision making result in a task that was expected to replicate the differences in endogenous oxytocin observed in that work. The Prisoner’s Dilemma used in this study consequently tested participants with both familiar and unfamiliar partners in dyadic interactions and presented substantial monetary incentives for participants to demonstrate calibrated trust in their partners with up to $60 USD lost as a consequence of each misplaced trust decision. This paradigm and design was expected to promote significant variation in both subjectively assessed trust/trustworthiness and endogenous oxytocin.

We therefore conducted two experiments designed to test existing oxytocin assays, assess baseline levels of human oxytocin in plasma, and then replicate and extend previous results reporting an association between peripheral oxytocin and trust or trustworthy behavior. In conducting these experiments, we sought to establish best practices for future studies involving oxytocin assays and address specific questions about the relationship between plasma oxytocin and trust/trustworthiness. In the first experiment, we used two of the most commonly used assays in published work, with and without extraction, and with and without 10 pg/mL of added, exogenous oxytocin. This value was chosen because it is at levels consistent with several published differential effect sizes such as the difference between autistic and non-autistic children [8] and the difference between mothers who do and do not receive epidural anesthesia during parturition [26]. Based on the results of the first experiment, we then selected an assay method and conducted the primary experiment which attempted to replicate previous work correlating oxytocin concentrations with trust or trustworthy behavior while observing the interaction between familiar individuals in the context of the face-to-face, monetary, Prisoner’s Dilemma paradigm. Twelve blood samples were drawn from each of 82 adults over the course of four rounds of the paradigm during one day. The choice to sample approximately every 30 minutes, in close association with decisions to trust or mistrust, was driven by the lack of data regarding release and clearance patterns for endogenous oxytocin, particularly in response to social interaction. This frequent sampling allowed us to test the responsiveness of plasma oxytocin to behavioral challenge in the task, check repeatability, and establish an overall normal population baseline. All assays were run in duplicate for sample concentration validation and averaged.

## Experiment One Methods: Comparison of Oxytocin Immunoassays in Human Plasma

### Ethics Statement

This study was reviewed and approved by the convened Institutional Review Board of the Air Force Research Laboratory (AFRL) in accordance with all applicable Federal regulations for the conduct of human subjects research.

### Participants

Thirteen participants (8 male) with a mean age of 31.4±9.4 years were recruited from an internal pool of AFRL employees. All completed comprehensive written informed consent prior to participation and reported no mental illnesses, substance abuse, or medication that would likely influence results. Participants were instructed to eat and sleep normally and abstain from alcohol, tobacco, and caffeine starting 12 hours prior to participation.

### Apparatus

Blood samples were collected via phlebotomy using a 21-gauge Safety-Lok blood collection set (“butterfly”; Becton-Dickinson, Franklin Lakes, NJ, USA) placed in the antecubital fossa. Plasma samples were collected using pre-chilled 6 mL K2 EDTA vacutainers (Becton-Dickinson, Franklin Lakes, NJ, USA) preloaded with protease inhibitor (100 µL of 0.76 mg/mL aprotinin in PBS, pH 7.4). We did note that the use of protease inhibitor may be unnecessary; Zhang et al. [17] demonstrated that oxytocin in plasma is stable for at least 17 hours at ambient temperature. A refrigerated centrifuge at 4°C was used for centrifugation, and samples were stored in a −80°C freezer. The enzyme-linked immunosorbent assay (ELISA) was the oxytocin kit from Enzo Life Sciences (Farmingdale, NY, USA). The radioimmunoassay (RIA) was the oxytocin kit from Bachem (Torrance, CA, USA) using an I^125^ label. RIA kits were used within two weeks of labeling in order to minimize losses of sensitivity due to I^125^ decay. Extractions were conducted using Oasis HLB 96-well plates, 60 mg sorbent per well, 60 µm particle size (Waters, Millford, MA, USA).

### Procedure

Following screening and informed consent, medical technicians placed the blood collection set in the antecubital fossa and immediately began blood draws. Approximately 3 mL of blood was drawn and discarded to ensure that circulating blood was collected in the subsequent samples. Eight vials were filled for a total of approximately 40 mL of whole blood drawn per participant. Vials were gently mixed, placed on ice, and immediately centrifuged at 1600 g for 15 minutes. Plasma was then drawn off from all eight vials and combined in a 50 mL storage tube that was frozen at −80°C.

Processing then proceeded with samples being split into aliquots for each of the study conditions. Exogenous oxytocin was added (spiked) to half the samples. This was accomplished by adding oxytocin acetate salt hydrate (Sigma, Saint Lois, MO, USA); 25 µL of 1 ng/mL stock solution of oxytocin in PBS was added to 2.5 ml of plasma or serum. If used, extractions were performed using the Oasis 96-well plates. Samples were then lyophilized and dissolved in 250 µL of assay buffer. Each of the two replicates used100 µL of the resulting solution. Unextracted samples were neat plasma also at 100 µL per assay. The remaining steps of the assay were conducted according to kit instructions.

## Results

**Figure 1:**
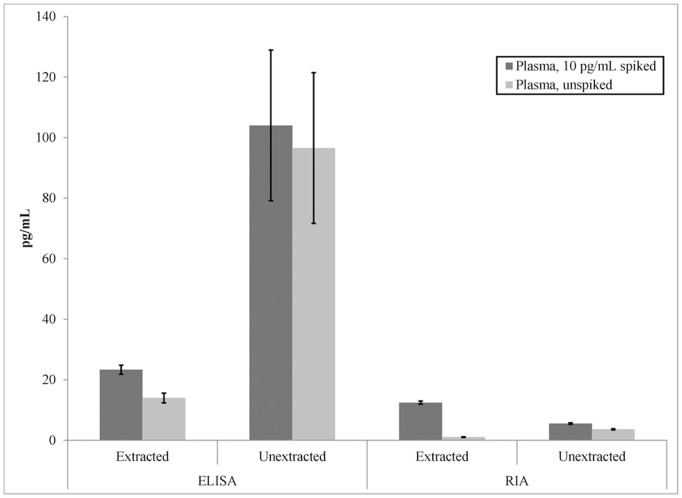
Oxytocin assays of human plasma. Mean reported concentrations of oxytocin and +/− one standard error of the mean for each of the conditions calculated across 13 participants. “Spiked” refers to those samples that had 10 pg/mL of oxytocin added, while “unspiked” reports the results of assaying an unspiked aliquot.

The ELISA kit exhibited high sensitivity to extraction (Fig. 1). Enzo Life Sciences recommends that sample extraction be performed with plasma; these were the only samples with acceptably low variance; the coefficients of variation (CVs) between duplicate samples confirm this observation. Without extraction, the mean CV was 20.4±3.7%, while with extraction, the CVs averaged 3.8±0.9%. The RIA kit did not exhibit similar sensitivity to extraction; however, as will be explored in the next section, it did not accurately recover the exogenous spike without extraction. CVs for the RIA averaged 4.1±0.8% with extraction and 2.9±0.5% without. The lower limit of quantitation (LLOQ), as published with the kit, is 0.6 pg/mL for the RIA and 11.7 pg/mL for the ELISA. With extraction, samples were concentrated approximately ten fold, resulting in valid oxytocin measurements as low as 1.1 pg/mL for the ELISA kit and 0.08 pg/mL for the RIA. We chose not to perform a full ANOVA on these data due to the large differences in results between the ELISA and RIA. A repeated-measures analysis was used as each participant contributed to all cells of the design (See statistical rationale in Text S1 in S1 File for further details). A 2×2 (spiked versus unspiked, extracted versus neat plasma) repeated-measures ANOVA was first conducted on the ELISA data. This analysis revealed no main effect of spiking, F(1,12) = 1.65, p>0.05, however, there was a main effect of extraction, F(1,12) = 8.08, p = 0.015. Applying the same ANOVA to the RIA results, we observed significant main effects for both factors. There was a significant main effect of spiked versus unspiked, F(1,12) = 496.57, p<0.01. There was also a main effect of extracted versus unextracted, F(1,12) = 10.81, p = 0.006 and a significant interaction between spiking and extraction, F(1,12) = 314.01, p<<0.01. The interaction is due to attenuation of the detection of the spike in unextracted samples; spiked samples were always at higher concentrations than unspiked samples.

**Figure 2:**
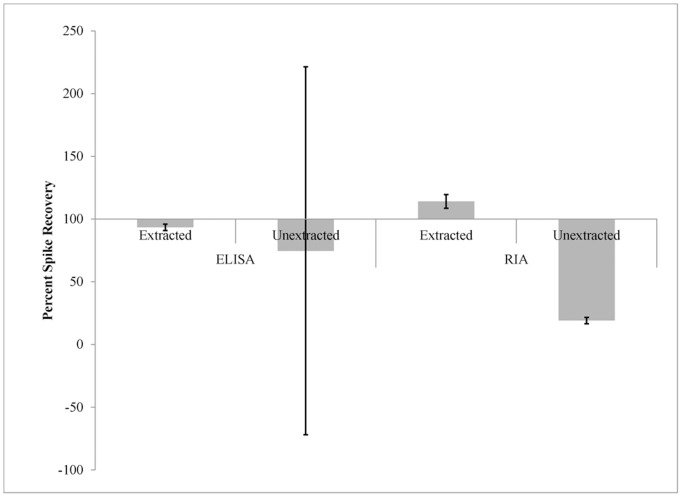
Spike recovery. Recovery of the 10 pg/mL spike, calculated as the percent difference between spiked samples and the paired, unspiked samples. Error bars are +/− one standard error of the mean across 13 participants.

By subtracting unspiked samples from corresponding spiked samples and dividing by the expected value of 10 pg/mL, we obtained a spike recovery percentage. If the spike was correctly delivered and the assay is accurate and precise, we would expect 100% recovery consistently. If the extraction step filtered out biologically active oxytocin, we would expect recovery values below 100%. Recovery for all conditions is plotted in Fig. 2. In order to test for systematic error (consistent deviation from the expected 100%), sign tests were conducted for each condition based on positive or negative difference from 100%. Statistical significance with this test implies that the sample mean is significantly different from the expected value. The sign test was chosen in order to minimize assumptions and simply test if the distribution of recovery percentages was centered on the expected 100%. For the ELISA, extracted samples were significantly different from the expected recovery, with 10 of 13 participants less than 100%, p = 0.044 (Bonferroni corrected for four comparisons); however as may be seen in Fig. 2, the values were very close to 100%. For the RIA, recovery in extracted samples was close to 100%, with 9 of 13 participants greater than 100%, p = 0.184. Without extraction, the ELISA produced highly variable results; the sign test indicated that the mean recovery was less than 100%, with 10 of 13 participants assayed less than 100%, p = 0.044. Lastly, the RIA without extraction produced uniform recovery values below 100%, p<0.001. All of the recovery values in this condition were well below 100%, indicating consistent underreporting of the spike. To quantify the impact of assay variance on research protocols, a power analysis was conducted using G*Power 3 [27]. To maximize power, the analysis was conducted for a paired t-test, with the observed sample variance used as the population variance for each assay with and without extraction. For the ELISA without extraction, this procedure estimated that a minimum of 28 participants would be required to detect a 10 pg/mL effect at *p*<0.05. Similar analyses for either assay with extraction suggest that as few as three participants are sufficient to achieve results statistically significantly different from zero given a constant effect.

**Figure 3:**
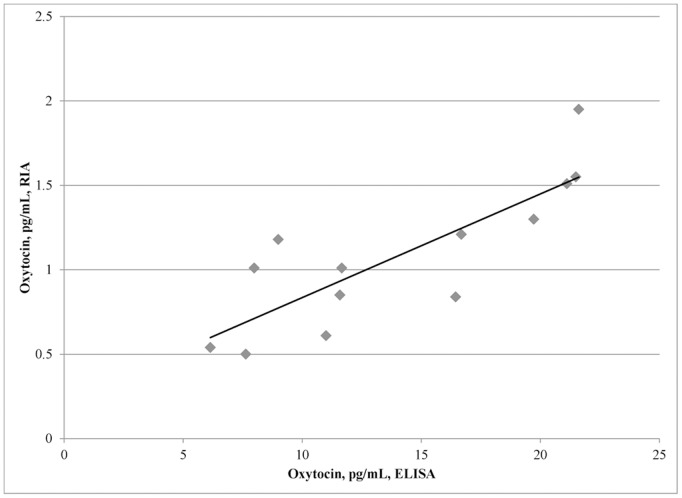
Comparison of ELISA-assayed oxytocin concentrations with RIA-assayed concentrations for extracted plasma samples. Each point is a single participant; aliquots for each assay were drawn from a within-participant pool. The plotted line is the line of best fit via linear regression.

On the basis of this last analysis, we would judge extracted plasma assayed by either the ELISA or the RIA to be good choices of methods and medium. However, comparing individual participants in just the extracted plasma condition, the RIA result correlates only moderately well with the ELISA result, at R^2^ = 0.67, though this is statistically significant, t(11) = 4.76, p<.001 (Fig. 3). Additionally, even with extraction, the concentrations reported by the two assays differ by an order of magnitude, with ELISA reported concentrations approximately 16 times greater than RIA. These data may also be used to infer a normal baseline and range: for the ELISA, the mean concentration was 14.0 pg/mL, with a standard deviation of 5.7 pg/mL, while the corresponding values for the RIA were 1.1 pg/mL and.4 pg/mL. Three standard deviations below either mean results in a minimum of zero (below LLOQ). Three standard deviations above these means results in a maximum of 31.1 pg/mL and 2.3 pg/mL, respectively. Conservatively, we may state that observed baseline values above 40 pg/mL are suspect; the highest assayed unspiked oxytocin concentration of the 13 participants was 21.6 pg/mL with the ELISA. As Zhang et al. [17] reported endogenous concentrations consistent with the values produced by the RIA, we chose to conduct the second experiment using extracted plasma assayed using the RIA. In this second experiment, we attempted to replicate natural variation in blood plasma oxytocin associated with trusting and trustworthy behavior in a monetary decision-making game as well as differences when interacting face-to-face with a familiar, as opposed to an unfamiliar, task partner.

## Experiment Two Methods: Endogenous Oxytocin in a Prisoner’s Dilemma Paradigm

### Prisoner’s Dilemma Task

**Figure 4:**
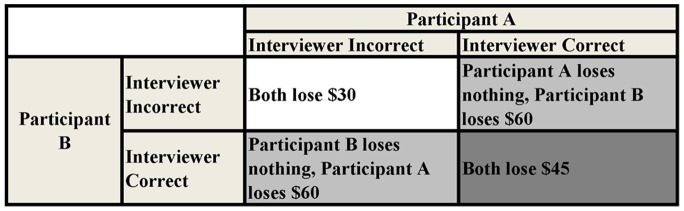
Payoff matrix used in the iterated Prisoner’s dilemma. All losses were removed from an initial endowment of $120 USD per partner pairing, and each partner pairing consisted of two rounds of the Prisoner’s Dilemma task. Any funds remaining from the sum total of the two endowments ($240 USD) at the end of the study were added to the participant’s hourly compensation.

The basic Prisoner’s Dilemma is a dyadic decision making task in which each participant must decide to cooperate or defect (analogized as prisoners accused of a crime choosing to remain silent or testify against his/her partner) with monetary payouts dependent on the joint outcome. This basic task was modified from the structure presented in Wedekind and Milinski [28] by presenting the aggregate payouts as losses from an endowment to maximize perceived value (e.g. [29]), multiplying payoff point values by a factor of 15, using United States Dollars (USD) instead of points, and decreasing the incentive to cooperate by reducing the effective payout when both participants cooperated (Fig. 4). This task also included a small probability that a decision to cooperate could be uncontrollably converted to a defection (further details in *Procedure)*; this was intended to give participants a plausible cover story to hide their own untrustworthy behavior from their partners.

### Participants

This study was reviewed and approved by the Air Force Research Laboratory (AFRL) IRB in accordance with all applicable Federal regulations for the conduct of human subjects research. A total of 120 naïve participants (53 male) with a mean age of 28.0±7 years were recruited from the general population in Dayton, Ohio and completed comprehensive written informed consent. All reported normal or corrected-to-normal vision, and no mental illnesses, substance abuse, or medication that would likely influence biomarker results. Participants were recruited in pairs with a familiar friend or family member with whom they already had a trusting relationship. Participants were instructed to eat and sleep normally and abstain from alcohol, tobacco, and caffeine starting 12 hours prior to participation. Participants received monetary compensation at an hourly rate in addition to keeping any of their endowment not lost in the course of completing the Prisoner’s Dilemma task.

### Apparatus and Stimuli

Pencil and paper surveys were used for all standardized questionnaires. Participants completed baseline measures of relationship closeness to their familiar partner (Relationship Closeness Inventory, RCI) [30] and repeated measures of trust state (Mayer three-factor trust inventory for ability, benevolence, and integrity, Mayer ABI) [31] and state-based anxiety (State-Trait Anxiety Inventory, STAI) [32]. The choice to cooperate or defect was instantiated with the use of 10 identical, small, numbered plastic boxes arranged in a two-by-five array with total dimensions of approximately 30×7.5 cm. During a face-to-face, dyadic interaction, participants were instructed to read a moral dilemma, write their answer to the dilemma on a small slip of paper, and then hide the slip of paper in one of the ten boxes. Desktop PCs were used to display post-round questions and the outcome of the partner’s interview using custom software implemented in MATLAB R2010 (The Mathworks, Inc.; Natick, MA, USA).

Blood samples were obtained via peripheral venous catheter placed in the antecubital fossa. Sterile saline solution was injected into the catheter following sample collection points in order to prevent obstruction. Prior to each sample collection, approximately 3 mL of fluid was drawn into a waste tube in order to ensure that no saline diluted the samples. All other procedures replicated those used for the RIA with sample extraction in Experiment One. All samples were run in duplicate, with average concentration used in subsequent analyses.

### Procedure

Participants first completed a telephone screening that was designed to identify persons who had a diagnosed mental disorder, who were on psychoactive or other medication that could influence results, or who were in poor health. Participants who did not self-report any of these exclusion criteria were then scheduled for testing and instructed not to consume alcohol, tobacco, or caffeine 12 hours prior the beginning of the study. Although there is evidence that there is no variation of oxytocin throughout a 24-hour cycle [33], all participants started the study at 8∶30 AM. After viewing an informational video, participants completed comprehensive written informed consent. They then watched a second video with task instructions (see Text S2 in S1 File for full instructions) and were individually fitted with peripheral venous catheters. After completing a short practice session and the RCI, participants earned their monetary endowment ($120 USD) with a sham survey-answering task. Participants then met with their first partner (either familiar or unfamiliar, randomized for each day of testing) to complete a moral dilemma (the Trolley or Footbridge Dilemmas [34]), write down their answers, and jointly select a box number in which they would both hide their secrets. Each participant had his or her own set of boxes and held on to it throughout the day. Partners also had the opportunity to discuss any strategy they may wish to use for completing the Prisoner’s Dilemma task (such as agreeing to jointly cooperate, jointly defect, etc.). Partners were then split up and individually asked by an experimenter if they wished to disclose the location of the secret. Since deception of the experimenter was not permitted, any “yes” response was treated as defection. If a participant chose not to disclose the location, the experimenter guessed one of the ten boxes. A correct guess was then treated as if the participant had defected. This was intended to provide an opportunity for participants to deceive their partner about the reason for the outcome of the interview (i.e., to hide untrustworthy behavior by claiming the experimenter interview resulted in a correct guess). After the experimenter opened a box, the custom software displayed text on an adjacent monitor indicating whether their partner’s experimenter identified the correct box, but did not clarify whether this was a consequence of defection or a correct guess. Participants were then reunited with their partner to discuss the interview outcome and review or change strategy, if they wished, prior to completing a second round. Participants were provided lunch and partners were then swapped (if familiar first, then unfamiliar after lunch, and vice versa). Each participant then completed two additional rounds with their new partner.

**Figure 5:**
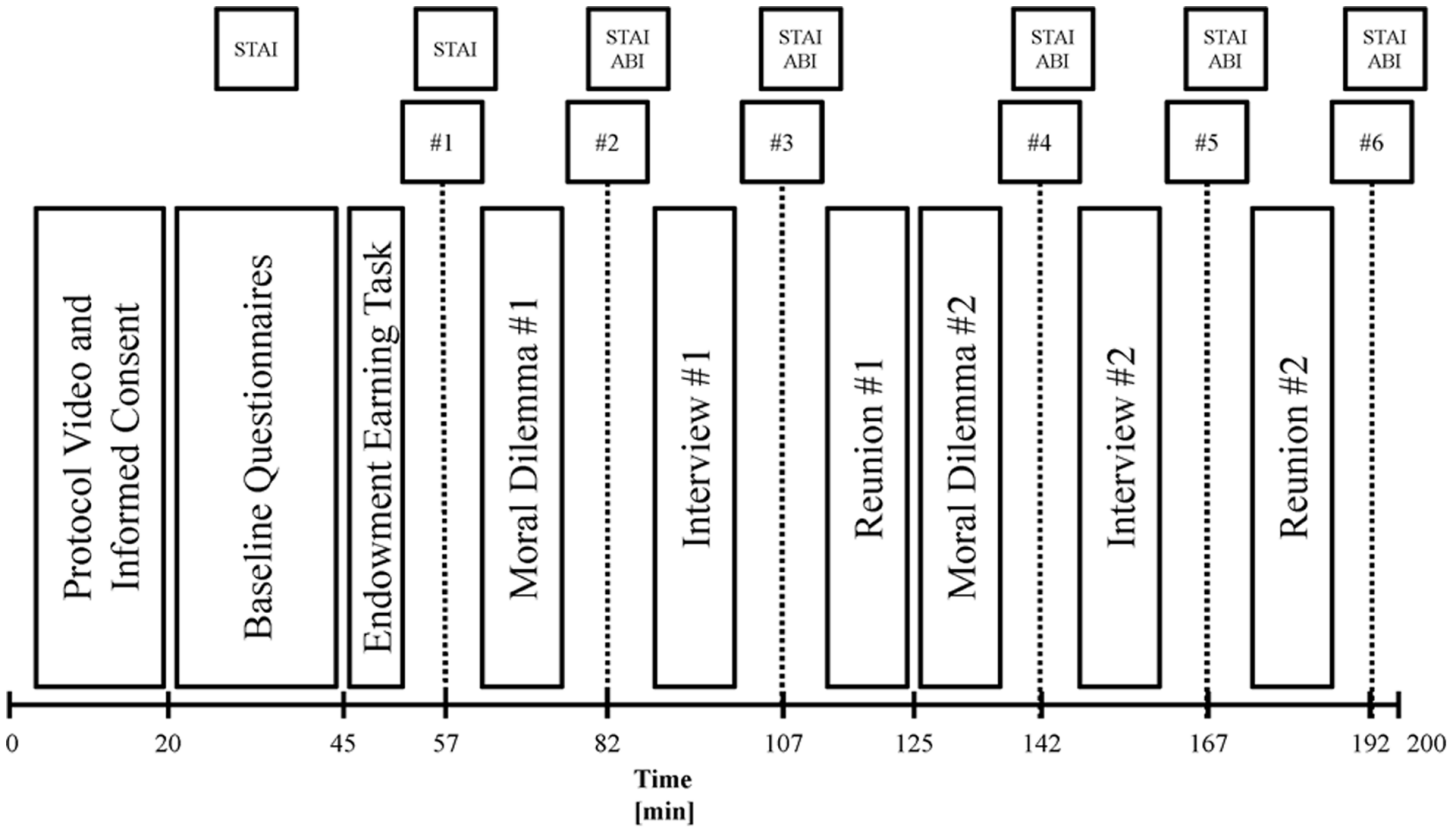
Timeline of blood draws and experimental stages for the first half of Experiment Two. The numbered boxes correspond to blood draws, while STAI and ABI are the subjective inventories. Timing between stages is somewhat approximate as there was minor variation in the time for different participant dyads to complete each stage. The second half of this experiment repeated this process following lunch and a switch in task partners.

Blood samples, the Mayer ABI, and the STAI were collected approximately every 30 minutes: 5 minutes before and 2 minutes after each 10 minute interview/decision point, with approximately 15 minutes in-between rounds (Fig. 5). Baseline blood samples and STAI were collected at the beginning of each session (morning and afternoon) prior to any partner interaction. No baseline Mayer ABI was collected as participants had not met their unfamiliar partners and thus could not answer meaningfully without prior interaction.

## Results

The first 60 participants in the study participated with both their familiar partner and an unfamiliar partner, in random order. Following early results that showed zero defections (mistrusting/untrustworthy behavior) in familiar pairs, the familiar partner interaction was replaced with a second unfamiliar partner interaction for the next 60 participants. All comparisons of trust and trustworthiness are drawn from only unfamiliar interactions. Twenty participants did not complete the full testing day due to various technical problems; those participants were excluded from analysis. The results presented here are from the remaining 100 participants. Blood samples were successfully drawn from 82 of these 100 participants.

Behavioral results indicated a very high proportion of trusting decisions. Due to the symmetric nature of the task, trust and trustworthiness are not clearly distinguishable; a participant may choose to adhere to a promised strategy because they trust their partner or because they are generally trustworthy. As we cannot determine precise motivations, for the purposes of this manuscript we will adopt an egocentric view and refer to a participant’s own behavior as trusting, and their partner’s behavior as trustworthy. Trusting decisions were coded by reviewing the participants’ strategies rather than cooperate or defect decisions. Behavior was scored as trusting when participants adhered to the strategy that they had agreed upon with their partner, with non-adherence scored as mistrusting. Similarly, partner trustworthiness was scored based on their partner’s adherence (trustworthy) or non-adherence (non-trustworthy) to the strategy. In 370 of 400 completed rounds (92.5%), the agreed-upon strategy was to not reveal the location of the secrets to the experimenter, thus making trusting behavior and cooperation nearly synonymous in this study. Twenty-one participants mistrusted once, 11 mistrusted more than once, and 68 never mistrusted, resulting in a 12.5% mistrust rate on a per-round basis. None of these mistrusting rounds occurred when interacting with a familiar partner.

**Figure 6:**
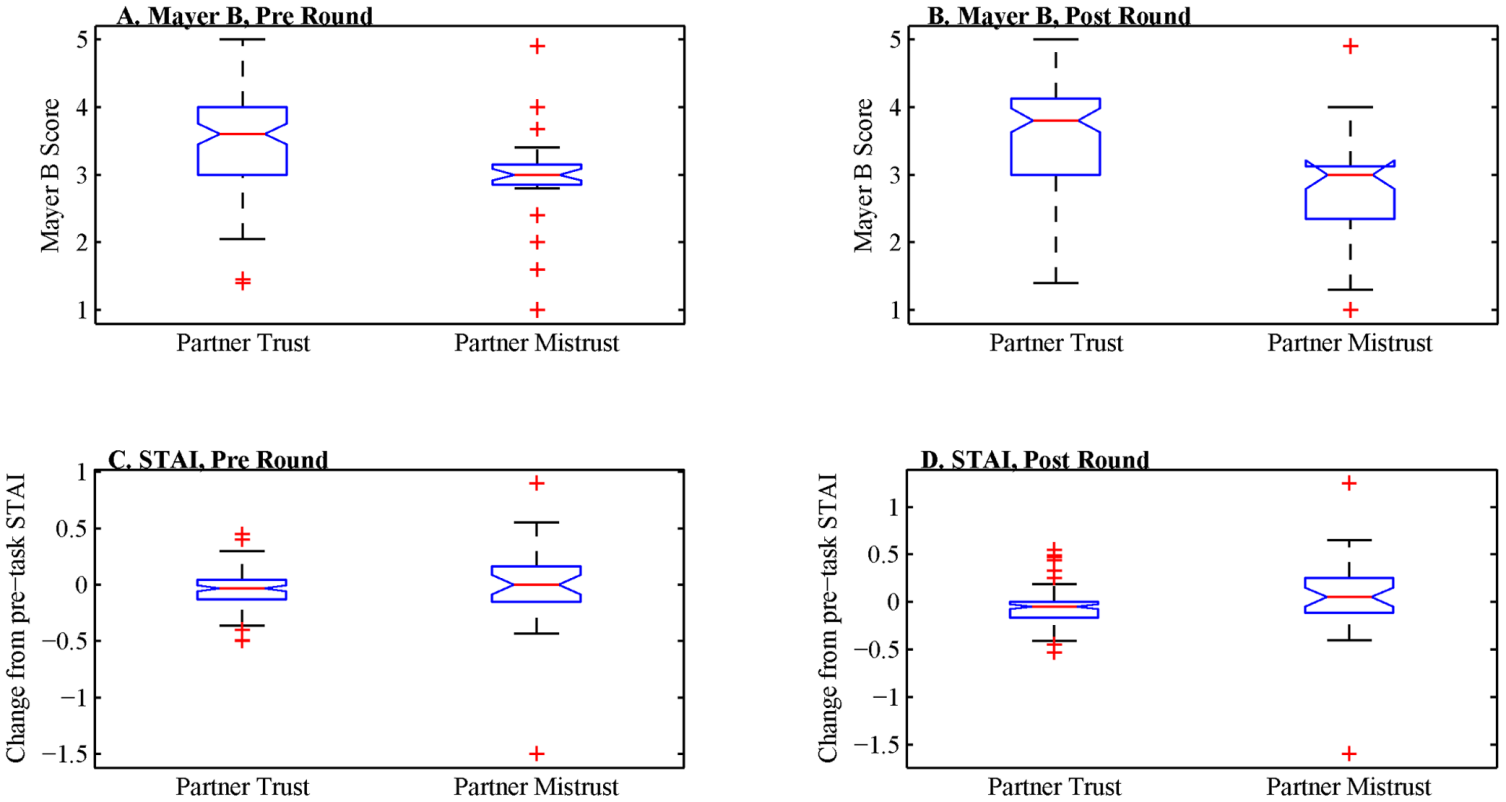
Distributions of subjective inventory scores during the task as a function of partner behavior. Panels A and B depict the distribution of Mayer Benevolence scores before and after a round of the task, respectively. In this figure and the following, boxes plot the median and inner quartiles with the whiskers extending to three standard deviations above and below the median. Any outlier values beyond this range are plotted as crosses. The notched sides represent the 95% confidence interval about the median.

The RCI scores confirmed that the familiar partners were indeed close with a mean total RCI of 9.7 and SD = 3.9, which was within one standard deviation of the 12.2 score reported for the closest relationship by Berscheid, Snyder, and Omoto [30]. The Mayer ABI and STAI scores collected before and after each round were divided into two groups and compared: the scores of those participants whom demonstrated trust and those whom demonstrated mistrust in their partner. Alternatively, the same scores were also divided and compared according to their partner’s trustworthy or untrustworthy behavior during the round. Fig. 6 shows the distributions of Mayer Benevolence and STAI before and after each round. The three subscales of the Mayer ABI were highly intercorrelated (all r>.8); consequently, only distributions for Benevolence are shown in detail. In this and all subsequent analyses, participants with samples more than three standard deviations above or below the mean were excluded; at most three participants were removed by this criterion. If a participant made multiple trusting or mistrusting decisions, those scores were averaged; each participant thus contributed one value for trust and one for mistrust in order to avoid overrepresentation of a subset of participants. Similarly, multiple examples of experiencing trustworthy or untrustworthy behavior from a partner were averaged. To maximize available data, these tests were conducted first as independent *t*-tests and then as paired *t*-tests for those participants with examples of both trust and mistrust (likewise trustworthy and untrustworthy partners), with False Discovery Rate procedures used to control for multiple comparisons [35] and Levene’s test for inequality of variance and corrected *df*. The full results of these tests are presented in the Tables S2 and S3 in S1 File. However, to summarize, we observed that the Mayer ABI varies significantly with both trust in a partner and partner trustworthiness, whether sampled before or after an interview/decision point (all *p*<0.05, corrected). The STAI varied primarily with a participant’s trust in their partner, either before or after a round; we also observed that subjective anxiety as measured by the STAI increased following a round in which a partner demonstrated untrustworthy behavior. There was no significant difference in subjective anxiety before a round as a function of that partner’s later trustworthiness. These results demonstrate that the task was successful at inducing significant variation in subjectively assessed trust in and trustworthiness of an unfamiliar partner, both before and after a round of the task, while subjective anxiety was associated with trust behavior and reactive to but not predictive of partner trustworthiness.

The 82 participants that had successful blood draws contributed up to twelve samples each taken 30 minutes apart over the course of the experimental task. Figure S1 in S1 File plots the overall distribution of concentration values obtained from these participants. We observed no significant effect of time of day (Figure S2 in S1 File), nor did we observe a significant difference in oxytocin following a supplied midday meal. All but two participants produced maximum plasma concentrations less than 10 pg/mL. Consistency was also good between duplicate assays, with a mean coefficient of variation of 3.7% (Figures S3 and S4 in S1 File). The overall observed range was 0.06 to 52.56 pg/mL, with a mean of 1.16 pg/mL and standard deviation of 3.55 pg/mL. The mean value is consistent with both the unspiked samples assayed with identical methods in Experiment One and the results of Zhang et al. [17] which were obtained without immunoassays.

**Figure 7:**
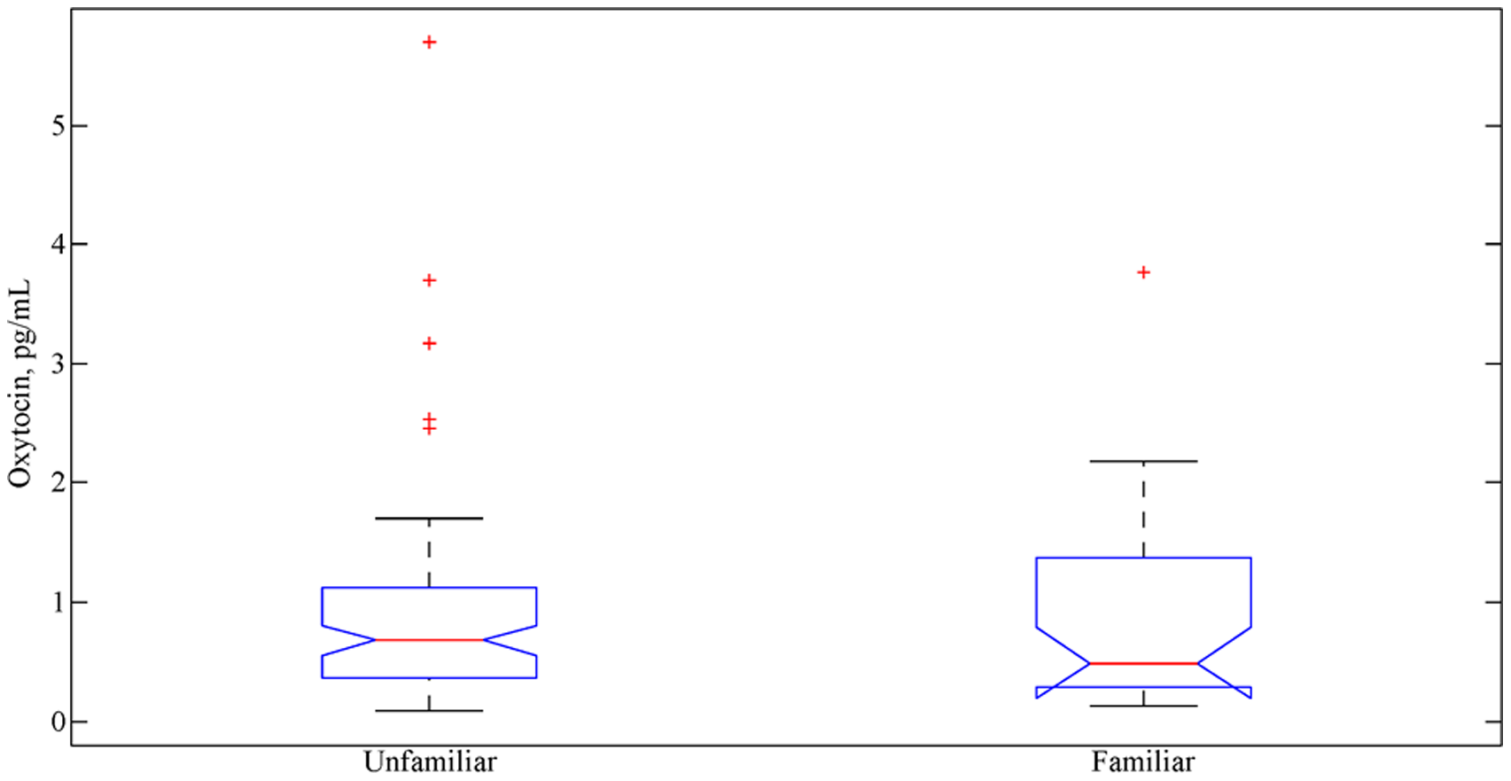
Distribution of oxytocin concentrations as a function of interacting with a family member or familiar versus an unfamiliar partner. Thirty-three and sixty-four participants are included in the familiar and unfamiliar distributions, respectively, due to the protocol change that replaced the familiar interaction with a second unfamiliar interaction.

**Figure 8:**
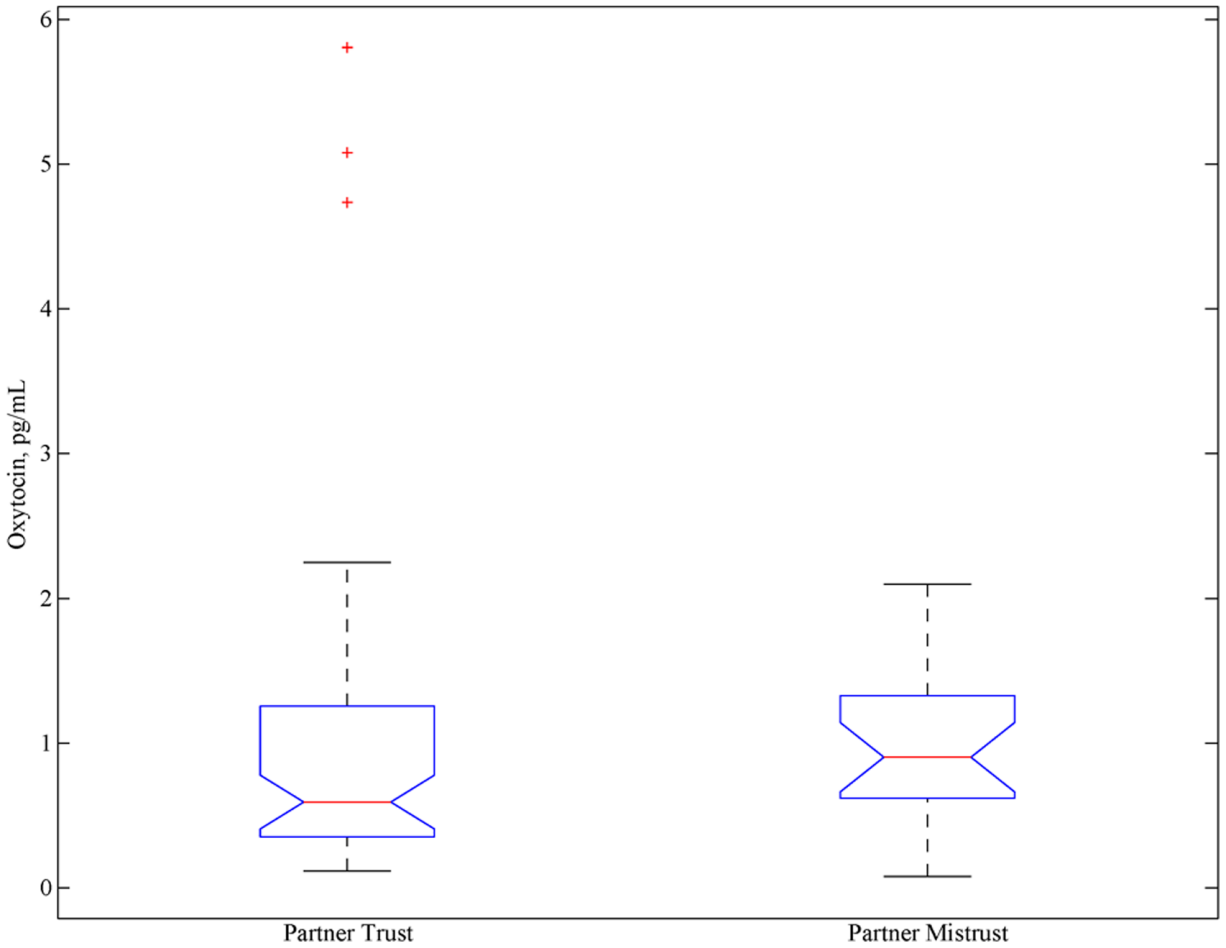
Distribution of oxytocin concentrations as a function of having a partner who demonstrated trust or mistrust in a participant. Twenty-two and sixty-four participants are included in the mistrust and trust distributions, respectively, due to the preponderance of trusting behavior as compared to mistrusting behavior.

The effects of the familiarity manipulation and trustworthiness on oxytocin in the Prisoner’s Dilemma task are plotted in Figs. 7 and 8. Fig. 7 compares oxytocin concentrations approximately 5 minutes following a face to face interaction with a familiar friend or family member as compared to those obtained following interaction with an unfamiliar individual. Interactions with a familiar individual were compared to unfamiliar interactions by averaging all samples taken during such interactions for each participant such that each participant contributed at most one value for familiar and one for unfamiliar interaction. Participants with samples from both interactions were first submitted to paired t-tests with no correction for multiple comparisons; these tests were run with raw values, proportions relative to baseline, and difference from baseline (subtracted). The smallest *p-*value observed was with individual baselines subtracted, *t*(28) = 1.2, *p* = .23 (two-tailed). In order to use all available data, independent-samples t-tests were also run. This produced very similar results; the smallest *p-*value was again with individual baselines subtracted, *t*(56) = 1.01, *p* = .31 (see Table S4 in S1 File for full results). Despite attempting to maximize statistical power, we did not observe significant differences in oxytocin concentrations between familiar and unfamiliar interactions.

The analysis of trust was motivated by Zak, Kurban, and Matzner [25] who reported significant differences in oxytocin values prior to a participant’s own decision to trust or mistrust and also reported a post round relationship between oxytocin concentrations and funds returned to a partner. Fig. 8 compares oxytocin concentrations following a partner-interaction: the oxytocin concentrations for participants whose partners demonstrated trustworthiness is compared to the concentrations for participants whose partners did not demonstrate trustworthiness. As with the familiarity manipulation, the oxytocin concentrations from multiple examples of trustworthiness or untrustworthiness for each participant have been averaged; each participant thus contributed at most one value for trustworthiness and one for untrustworthiness. In order to provide strong evidence that there is no effect, we again utilized both paired and independent t-tests of oxytocin values. As may be inferred from Fig. 8, there was no significant difference in post-round oxytocin as a function of partner trustworthiness, *t*(82) = .31, *p = *.75. These data were also analyzed prior to the decision point/interview, and expressed as proportional or absolute difference from pre-task baseline; in all cases, these comparisons were not significant. The effect of a participant’s decision to trust his/her partner was also examined in this fashion; no significant results were obtained. The lowest observed *p*-value was for a paired test of raw oxytocin concentrations, prior to experiencing trust or mistrust from a partner, *t*(20) = 2.69, *p = *.014; however, this result is not significant after correcting for multiple comparisons using either the Bonferroni procedure or correcting via FDR [35] (see Table S5 in S1 File for full results).

## Discussion

The literature review, the upper bound of oxytocin concentrations obtained with the ELISA after extraction in Experiment One, and the largest observed values with the RIA in Experiment Two all suggest that reported normal baseline oxytocin values in human blood averaging above approximately 40 pg/mL are unreliable. Roughly half of the studies we identified that assayed human blood plasma samples collected from the periphery reported values higher than 40 pg/mL, all of these higher values were observed when sample extraction was not performed. We consistently produced values larger than this only with the use of the ELISA kit without extraction, which resulted in poor CVs and high variance across participants. Of the 752 samples assayed with extraction in Experiment Two, only three samples (0.3%) from one participant fell near or above 40 pg/mL. Both the ELISA and RIA kits tested here are sensitive to extraction and produce inconsistent results when samples are not extracted. This is consistent with results obtained by Szeto et al. [14]. However, that work used an RIA with insufficient sensitivity to detect normal circulating levels, thus no direct comparison with the ELISA kit was possible.

Spike recovery was accurate for both assays from extracted plasma only. Based on these results, either the RIA or the ELISA kits are acceptable assays as long as extraction is performed, though the relatively poor correlation between the two methods is worrisome. We prefer the RIA for two reasons: it is capable of quantifying oxytocin at concentrations approximately 10 times lower than the ELISA, and the resulting concentrations are consistent with our expectations for accurate baseline values in plasma.

Our results suggest that the correct normal human baseline range for endogenous oxytocin is 0–40 pg/mL, although the results obtained via RIA with extraction suggested a range of approximately 0–10 pg/mL; in Experiment Two, 99.5% of samples fell within this range. Using similar methods, other research groups have published values in this range (e.g. [36], [37]). Zhang et al. [17] observed concentrations consistent with the RIA with extraction (∼1 pg/mL) using costly and time consuming, but specific and precise methods. Their 2D LC-MS/MS method does not rely on antibody binding as in the immunoassays and was demonstrated to be capable of similar LLOQs to the RIA, though the cost and complexity of the method will limit widespread usage. This converging evidence in favor of the lower range for oxytocin motivated the use of the RIA with extraction for the second experiment. Regardless of the methods chosen, researchers should be explicit when reporting methods in publishing oxytocin assay results as the expected range of values significantly depends on the method used.

In our second experiment, we did not observe statistically significant associations between plasma oxytocin and trust, trustworthiness, or familiar interaction. Based on our methodological testing, our methods are sufficiently precise to detect small changes in oxytocin levels; the observed standard error across 13 participants in Experiment One was 0.12 pg/mL, equal to or less than the size of effects previously published for behavioral challenge paradigms. One possible explanation is that the trust-related effects associated with these manipulations are primarily central and, consequently, peripheral effects are dependent on transfer from CSF to plasma which is known to be very limited [16]. Another possibility is that our sampling points did not properly target the pulsatile release of oxytocin if, indeed, oxytocin release in interpersonal interaction is pulsed as it is in parturition and lactation [38]. Such a release may be very brief; the half-life of infused exogenous oxytocin is known to be no more than 3–4 minutes [39], [40]. However, release and clearance patterns for endogenous oxytocin associated with psychological phenomena are generally unknown. While our sampling was close to or within this 3–4 minute half-life, greater precision in sample timing could increase experimental sensitivity if these factors were well-understood. Even with this knowledge, obtaining precise control over the associated behavior may render appropriately synchronizing blood draws difficult.

It is also possible that the manipulations tested here do not cause humans to release detectable amounts of oxytocin. In creating the experiment, we went to considerable lengths to increase the participants’ sense of consequence and concreteness during the experimental task over previously published work and closely link sample points to key decision events. Significant differences in self-assessed trust were observed, indicating that the experiment was successful in producing meaningful variation in trust. Thus, we consider it unlikely that significant peripheral releases of oxytocin are associated with trust or trustworthy behavior in this type of task.

Our first study revealed that both the ELISA and RIA kits are sensitive and capable of accurately recovering a 10 pg/mL spike in an appropriate medium: extracted plasma. Unfortunately, the correlation between the two kits when used for endogenous measurements under these conditions is only moderately high at R^2^ = 0.67, and there is a considerable scalar difference between the two. The scalar difference could reflect differing cross-reactivity with closely related molecules, such as the prohormone or breakdown products, differences in sensitivity to matrix interference, or any of a number of similar causes. However, this scalar difference may be of little consequence in studies that examine only relative concentrations of oxytocin. In our studies, we were concerned with absolute concentrations as well as relative concentrations and therefore, preferred the RIA kit.

## Limitations

The present behavioral study (Experiment Two) sampled blood plasma from the periphery in an attempt to detect an association between oxytocin and trusting or trustworthy behavior. From the results of Experiment One, the assay used demonstrated good sensitivity, however there are many unknowns that limit these results. The exact time course and nature of oxytocin release in social interaction paradigms is unknown; if release was brief and pulsatile in response to face to face interaction, then oxytocin levels may have returned to baseline prior to our sampling. The paradigm used does not permit highly precise coupling of oxytocin sampling to behavioral events; lacking an objective means for localizing an internal decision to trust, sampling was keyed to the observable behavior of displaying trusting behavior. Our work does not address central oxytocin levels; it is likely that oxytocin in CSF is not coupled to oxytocin in the periphery. Similarly, the assay used is targeted to biologically active, amidated oxytocin; it is possible that other species may exhibit some affinity for oxytocin receptors and thus the assay does not fully reflect biological activity.

Due to the symmetric nature of the task, we cannot separate a decision to trust one’s partner from a decision to behave in a trustworthy fashion. Likewise, participants may have chosen to adhere to the agreed-upon strategy due to compliance with social norms or preservation of self-concept (e.g. [41]) rather than based explicitly on trust. Nevertheless, we observed statistically significant differences in subjectively assessed interpersonal trust and can thus conclude the task was successful at inducing significant variations in trust yet not oxytocin.

## Conclusions

Regardless of the immunoassay used, we strongly recommend performing solid phase sample extraction. Previous publications that have not used extraction with the ELISA kit should be interpreted with significant caution (e.g. [4], [5], [6], [25], [42], [43]). The CVs and recovery values we obtained indicate that this method of assaying oxytocin is unreliable. Although we confirmed that the RIA kit with extraction exhibited adequate recovery and CVs, we were not able to replicate previously published increases in peripheral oxytocin during interpersonal interactions involving trust. In future research on oxytocin in human plasma, we recommend first identifying a manipulation that stimulates oxytocin release and then densely sampling to estimate the kinetics of such release associated with observed behavior. Subsequent work should also examine the relevancy and biological activity of both oxytocin precursors and breakdown products, as a further step towards elucidating the appropriate analytes for future oxytocin research.

## Supporting Information

S1 File **Supporting text, tables, and figures.** This file consolidates the supporting text, tables, and figures. Text S1, Statistical Methods. Text S2, Participant Instructions. Text S3, Supplementary References. Table S1, Review of published human baseline oxytocin. Table S2, Experiment Two: differences in subjective trust pre- and post-decision. Table S3, Experiment Two: differences in subjective anxiety pre- and post- decision. Table S4, Oxytocin t-test results comparing familiar and unfamiliar Interactions. Table S5, Oxytocin t-test results comparing trust and mistrust. Figure S1, All oxytocin concentrations, by participant and sample number, in Experiment Two. Figure S2, Time course of average oxytocin concentration. Figure S3, Coeffecients of variance in Experiment Two. Figure S4, Mean concentration vs. variance in Experiment Two. Raw data used to generate figures and data tables may be found in S1 Data and S2 Data.
(DOCX)

S1 Data **Raw data from Experiment One.** This spreadsheet has all individual concentration values for Experiment One, split between RIA and ELISA results on individual sheets.
(XLSX)

S2 Data **Raw data from Experiment Two.** The individual sheets report values from before a round (pre) or after (post), with baseline not included (raw), proportion from baseline where available (div) or difference from baseline (sub). Trust behavior and partner trustworthiness are binary coded based on adherence to agreed-upon strategy, with 1 indicating adherence.
(XLSX)
