# Correction: Lack of Association between Human Plasma Oxytocin and Interpersonal Trust in a Prisoner’s Dilemma Paradigm

**DOI:** 10.1371/journal.pone.0119691

**Published:** 2015-03-19

**Authors:** The PLOS ONE Staff

The data columns in file S1_Data appear in the incorrect order. Please view the correct S1_Data file here.

## Supporting Information

S1 Data Supplementary dataset. This file includes supplementary data.
(XLSX)
