# Supplementary material for: Lack of Association between Human Plasma Oxytocin and Interpersonal Trust in a Prisoner’s Dilemma Paradigm
Source: PLoS One. 2014 Dec 30;9(12):e116172. doi: 10.1371/journal.pone.0116172 (PMC4280178; doi:10.1371/journal.pone.0116172)
Supplement: S1 File — Supporting text, tables, and figures. This file consolidates the supporting text, tables, and figures. Text S1, Statistical Methods. Text S2, Participant Instructions. Text S3, Supplementary References. Table S1, Review of published human baseline oxytocin. Table S2, Experiment Two: differences in subjective trust pre- and post-decision. Table S3, Experiment Two: differences in subjective anxiety pre- and post- decision. Table S4, Oxytocin t-test results comparing familiar and unfamiliar Interactions. Table S5, Oxytocin t-test results comparing trust and mistrust. Figure S1, All oxytocin concentrations, by participant and sample number, in Experiment Two. Figure S2, Time course of average oxytocin concentration. Figure S3, Coeffecients of variance in Experiment Two. Figure S4, Mean concentration vs. variance in Experiment Two. Raw data used to generate figures and data tables may be found in S1 Data and S2 Data. (DOCX) [file pone.0116172.s001.docx]

**SUPPLEMENTARY MATERIAL for LACK OF ASSOCIATION BETWEEN HUMAN PLASMA OXYTOCIN AND INTERPERSONAL TRUST IN A PRISONER’S DILEMMA PARADIGM**

**Text S1. Statistical Methods.**

*Experiment One*

There are three primary questions to be answered in analyzing the results of Experiment One:

1. What impact does sample extraction have on assay results?
2. Can any of these methods recover the spike accurately?
3. Do the ELISA and RIA methods produce consistent results for aliquots of the same sample?

Omnibus tests are generally preferred in order to minimize multiple comparisons, however including all of these factors in the same test results in an unwieldy, potentially uninterpretable three way test. Consequently, the first question was addressed via repeated measures ANOVA, separately for the ELISA and RIA. The ANOVA was selected in order to maximize power based on reasonable satisfaction of the basic assumptions of the test. This resulted in a 2 (extracted vs. unextracted) x 2 (spiked vs. unspiked) within subjects test. Levene’s test was used to adjust degrees of freedom in the presence of inequality of variances, however no such adjustment was found necessary.

The second and third questions were addressed following Szeto, et al. (2011). In their work, parametric and nonparametric methods were used to assess spike recovery percentages and correlation between expected and actual recovery and between the ELISA and RIA. We chose a nonparametric sign test of the spike recovery percentages to test accuracy; this minimizes assumptions made in regard to the sample distributions and presents a simple answer as to whether the distributions of spike recovery are centered on the expected value. The precision of the assays is reported as the coefficients of variation. There is no simple statistical test for acceptable precision, however we provided the coefficients and then attempted to give a practical sense of the implications by conducting a power analysis with our observed sample variance. Given the single spike quantity used, a correlation between actual and expected assay results is not a meaningful test, however correlating the results of the ELISA and RIA is informative and was tested for significance using the standard t-test as in Szeto et al. (2011).

*Experiment Two*

The methods used to analyze oxytocin data were intended to provide the most liberal, plausible tests of significant associations with plasma oxytocin and informed by prior work (e.g. Zak, Kurzban, & Matzner, 2005). Paired t-tests were selected as a powerful way to test for significant differences in mean oxytocin between two sets, though there are a number of assumptions made in regard to the sample distributions, and this limited testing to participants with examples of both behaviors. To maximize the number of participants in the test, we also conducted independent-samples t-tests. In keeping with previous work, we ran the tests on raw concentration values, but also included baseline-corrected measurements as either a proportion or simple difference. In an effort to be as complete as possible, we are reporting all tests run, though this creates a significant problem with multiple comparisons.

To address multiple comparisons given the large number of tests run, we have chosen to control the False Discovery Rate (FDR, Benjamini & Yekutieli, 2001) rather than Family-Wise Error (FWE). While a full discussion of this issue is beyond the scope of this article, by controlling FDR, we set a desired, acceptable rate of false discoveries (i.e. among only those tests that are significant, what proportion of false positives is acceptable) rather than controlling the FWE which seeks to control the probability of one or more false positives across all tests. Controlling FWE with larger numbers of tests can result in very conservative criteria that produce an unacceptably high rate of Type II errors.

**Text S2. Participant Instructions.**

Each participant viewed an instructional video to ensure that task instructions were delivered clearly and consistently. The script for this video follows.

PRESENTER: We call this task “Sharing Secrets”. You are first going to answer a few short questions – for each question you get correct, you’ll earn a significant amount of money. You’ll then switch to performing a different task, this time with your partner. In this task, you and your partner will come up with your individual answers to a tough moral dilemma, write them down, and then hide them in one of ten boxes. The location of your answers will be your secret to keep. You’ll then be interviewed separately about the box in which you hid the answer. Depending on how that goes, you may keep all the money you earned, lose it all, or something in between. You’ll get to keep any amount of money you have left at the end of the day.

PRESENTER: The interviewer will ask you a series of yes or no questions about which of the boxes holds the answer. Your interviewer is not a trained professional – they’re a regular person just like you and I. The interviewer will tell you that you will have the option to reveal the location of the secret when they point to it. The interviewer will then point to each of the boxes and ask you whether the secret is hidden in that box. If you decide not to reveal the location of the secret, you will answer “no” to each of their questions. The interviewer will then guess which box holds the secret. The interaction might go something like this:

INTERVIEWER (points to box #1): Is the answer in box #1?

PRESENTER: No.

INTERVIEWER (points to box #N, repeat for all 10 boxes): Is the answer in box #N?

PRESENTER (repeat for all 10 boxes): No.

INTERVIEWER: I’m going to guess that the answer is in box number 5.

PRESENTER: You also have the option to reveal the location of the secret. If you decide to reveal the secret, you should answer, “yes,” when they point to the box that holds the secret. The interaction might go something like this:

INTERVIEWER (points to box#1): Is the answer in box #1?

PRESENTER: No.

INTERVIEWER (points to box #2): Is the answer in box #2?

PRESENTER: Yes.

INTERVIEWER (points to box #N, repeat for all 10 boxes): Is the answer in box #N?

PRESENTER: No.

PRESENTER: As you can see, the secret may be revealed by you giving the secret to the interviewer OR the interviewer may just guess correctly. The interviewer will have a 10% chance of guessing the box correctly.

Depending on how the interviews go, you may lose none, a little, or a lot of the money you earned. If both you and your partner keep the secret, you’ll both lose a little money, but keep most of it. If you give up the secret, but your partner doesn’t, then you’ll lose nothing, and your partner loses a lot. However, the situation can be reversed: if your partner gives up the secret, and you don’t, you’ll lose a lot of money and they’ll keep all their money. Finally, if both of you give up the secret, you’ll both lose the same amount of money, which is more than you would lose if you both kept the secret. So if you think your partner might give up the secret, you may choose to give up the secret to try to keep as much money as you can. It all works like this:

PRESENTER: You and your partner can decide what to do, but if you’re going to keep the secret just say no to every question. If you both kept the secret (points) then you both get to keep most of your money, just losing $15 each. But, if your partner gave up the secret but you didn’t, then you lose $60 and your partner loses nothing. If you give up the secret but they didn’t, then you lose nothing and your partner loses $60. If you both give up the secret, you both lose $45. So you’ll have to think carefully about what your partner might do. You should also be aware that your partner may try to keep the secret, but they may not hide it well, or that the interviewer just guessed correctly. It may not be the case that your partner intended to give up the secret.

PRESENTER: After the interview is done, you’ll have a chance to come back into your individual testing room. You will then be shown the outcome of your partner’s interview while your partner finds out the results of your interview. This is presented only in terms of whether the interviewer selected the correct box. You will then be given the opportunity to talk with your partner about how the interview went. You will then repeat these steps and go through several rounds just like this. After all the interviews are over, we’ll see how much money you have left and then add that to your hourly pay. You will then be given a break for lunch before switching partners for the afternoon session. After all this is done, you’ll get a check for the full amount before you leave here today.

**Table S1. Review of published human baseline oxytocin.** This table collects published oxytocin assay results for baseline, non-pathological adult human plasma samples, unless otherwise noted. Under extraction, SPE refers to solid phase extraction.

|  | Extraction | Kit | Midpoint Value (pg/mL) | SD | Notes |
| --- | --- | --- | --- | --- | --- |
| Amico (1981) | None | RIA - Amico | 4 |  |  |
| Bagdy (1988) | None | RIA - Custom | 7 |  |  |
| Bello (2008) | None | ELISA - Enzo | 223 | 150 |  |
| Billhult (2008) | Ethanol | ELISA - Enzo | 23 | 11.2 |  |
| Borg (2011) | None | ELISA - Enzo | 160 |  |  |
| Borg (2008) | None | ELISA - Enzo | 200 | 133 |  |
| Carter (2007) | None | ELISA - Enzo | 175 | 25 |  |
| Chicharro (2001) | SPE-C18 | RIA - Phoenix | 0.17 | 0.02 |  |
| Cyranowski (2008) | Acetone | RIA - Amico | 3 |  |  |
| Domes (2010) | Vycor glass powder | RIA - Custom | 5.7 | 8.1 |  |
| Dumont (2009) | SPE-C18 | RIA - Custom | 0.8 | 0.3 |  |
| Feldman (2007) | None | ELISA - Enzo | 267.87 | 392 | Serum |
| Feldman (2010) | None | ELISA - Enzo | 365 | 138 |  |
| Floyd (2010) | None | ELISA - Enzo | 290 | 290 |  |
| Goldman (2008) | None | ELISA - Enzo | 240 | 224 |  |
| Gordon (2010) | None | ELISA - Enzo | 382 | 300 |  |
| Gordon (2008) | None | ELISA - Enzo | 258.76 | 38.41 |  |
| Gouin (2010) | None | ELISA - Enzo | 400 |  |  |
| Green (2001) | Acetone | RIA - Custom | 1.4 | 0.1 |  |
| Grewen (2005) | Acetone | RIA - Amico | 1.59 | 1.2 |  |
| Grewen (2008) | Yes – not specified | RIA - Bachem | 5.4 | 4.25 |  |
| Grewen (2010) | SPE - 33 um plate | ELISA - Enzo | 4.82 | 2.15 |  |
| Hoge (2008) | None | ELISA - Enzo | 145 | 52.9 |  |
| Holt-Lunstad(2008) | SPE - 33 um plate | ELISA - Enzo | 7.53 | 7.56 |  |
| Jansen (2006) | SPE - C8 columns | RIA - Custom | 5 |  |  |
| Keri (2009) | None | ELISA - Enzo | 240 |  |  |
| Lee (2003) | Acetone-ether | RIA - Custom | 2 |  |  |
| Light (2000) | Acetone-ether | RIA - Amico | 1.9 |  |  |
| Marazziti (2006) | SPE-C18 | RIA - Phoenix | 1.53 | 1.18 |  |
| Miller (2009) | None | ELISA-Other | 77.7 | 93 | Serum, R&D Systems EIA; Men |
| Miller (2009) | None | ELISA-Other | 33.5 | 37.7 | Serum, R&D Systems EIA; Women |
| Modahl (1998) | Acetone-ether | RIA - Custom | 1.16 | 0.77 |  |
| Nilsson (2009) | Ethanol | ELISA - Enzo | 73.4 | 11.3 |  |
| Ohlsson (2004) | SPE-C18 | RIA - Custom | 1.3 | 0.33 |  |
| Parker (2010) | Acetone | RIA - Amico | 1 |  |  |
| Péqueux (2001) | Membrane filtered (3 kDa) | ELISA-Other | 7.28 | 4.49 |  |
| Rahm (2002) | SPE-C18 | RIA - Custom | 42.3 | 23.5 | Women during active labor; for comparison only |
| Rubin (2010) | None | ELISA - Enzo | 322.93 | 187.05 |  |
| Salonia (2005) | SPE-C18 | RIA - Bachem | 2 |  |  |
| Scantamburlo (2007) | None | RIA - Custom | 3670 |  |  |
| Tabak (2011) | SPE-C18 | RIA - Phoenix | 1.61 | 2.78 |  |
| Taylor (2010) | None | ELISA - Enzo | 261.1 | 207.18 |  |
| Tops (2007) | Acetone-benzene | RIA - Custom | 50 | 9.6 |  |
| Turner(2002) | Acetone | RIA - Custom | 3.3 | 3.7 |  |
| Uckert (2003) | SPE-C18 | RIA - Bachem | 71 | 41 |  |
| Weisman (2012) | None | ELISA - Enzo | 375.78 | 264.03 |  |
| Wikstrom (2003) | SPE-C18 | RIA - Bachem | 1.8 |  |  |
| Wolff (2006) | SPE-C18 | RIA - Custom | 2.17 | 1.3 |  |
| Zak (2005) | None | ELISA - Enzo | 197.75 | 165.23 |  |
| Zhang (2011) | SPE (Oasis) | 2D LC MS/MS | 2.58 | 0.275 |  |

**Table S2. Experiment Two: differences in subjective trust pre- and post-decision.** Subjective trust (Mayer ABI) was compared using both independent-samples and paired *t* tests for task rounds in which the participant themselves (Self) demonstrated trust (by adhering to an agreed-upon strategy) vs. mistrust (not keeping with the agreed-upon strategy). Similarly, rounds in which the participants’ partner (Partner) demonstrated trustworthiness by adhering to the strategy vs. demonstrating untrustworthiness by not keeping with the agreed-upon strategy were also compared. These values were compared both before (Pre) and after (Post) each round. The Mayer ABI scores did not have a baseline, thus the tests were based on raw values. Degrees of freedom (df) vary due to variance in participant behavior and adjustments due to inequality of variance (via Levene’s test). The FDR procedure was applied to these tests as a whole; with q=0.05 and q=0.01, we obtained threshold *p*-values of 0.031 and 0.005, respectively. All of the Mayer ABI tests are significant, with all but one significant at q=0.01.

|  |  |  | **Independent** | | **Paired** | |
| --- | --- | --- | --- | --- | --- | --- |
|  |  |  | **df** | **p** | **df** | **p** |
| **Pre** | **Self** | **Ability** | 125 | .000 | 27 | .000 |
|  |  | **Benevolence** | 125 | .000 | 28 | .000 |
|  |  | **Integrity** | 127 | .000 | 28 | .000 |
|  | **Partner** | **Ability** | 131 | .031 | 32 | .002 |
|  |  | **Benevolence** | 131 | .000 | 32 | .000 |
|  |  | **Integrity** | 129 | .000 | 31 | .000 |
| **Post** | **Self** | **Ability** | 127 | .000 | 28 | .000 |
|  |  | **Benevolence** | 125 | .000 | 28 | .000 |
|  |  | **Integrity** | 127 | .000 | 28 | .000 |
|  | **Partner** | **Ability** | 131 | .001 | 32 | .001 |
|  |  | **Benevolence** | 131 | .000 | 32 | .000 |
|  |  | **Integrity** | 129 | .000 | 31 | .000 |

**Table S3. Experiment Two: differences in subjective anxiety pre- and post- decision.** In the same fashion as the ABI data (Table 2), STAI scores were compared. The scores were either left as raw values, expressed as proportion relative to baseline (Prop.), or simple subtractive difference from baseline (Diff.). The FDR procedure was applied to these tests as a whole; with q=0.05 and q=0.01, we obtained threshold *p*-values of 0.004 and 0.0001, respectively. Overall, we observed that self-reported anxiety is lower when a participant chooses to trust their partner than when they mistrust their partner (Self, Pre and Post). For the independent tests, this was observed only with baseline included, which suggests that individual differences in baseline anxiety can obscure this effect. With paired, within-subject tests, no such correction is necessary as baseline is presumably present in both paired values; as a result, it is unsurprising that all paired tests involving self-behavior are significant at q=0.05.

|  |  |  | **Independent** | | **Paired** | |
| --- | --- | --- | --- | --- | --- | --- |
|  |  |  | **df** | **p** | **Df** | **p** |
| **Pre** | **Self** | **Raw** | 42 | .104 | 28 | .002 |
|  |  | **Prop.** | 34 | .001 | 27 | .004 |
|  |  | **Diff.** | 35 | .000 | 28 | .002 |
|  | **Partner** | **Raw** | 131 | .313 | 32 | .329 |
|  |  | **Prop.** | 38 | .680 | 31 | .708 |
|  |  | **Diff.** | 39 | .086 | 31 | .101 |
| **Post** | **Self** | **Raw** | 42 | .085 | 27 | .004 |
|  |  | **Prop.** | 31.21 | .003 | 27 | .003 |
|  |  | **Diff.** | 32.33 | .001 | 27 | .002 |
|  | **Partner** | **Raw** | 131 | .083 | 32 | .028 |
|  |  | **Prop.** | 36 | .097 | 31 | .115 |
|  |  | **Diff.** | 39 | .086 | 31 | .027 |

**Table S4. Oxytocin t-test results comparing familiar and unfamiliar Interactions.** Similar to the analysis of trust (main manuscript, Figure 8), samples obtained during familiar interactions were compared to those obtained during unfamiliar interactions. This comparison was first limited to those participants with both interactions, via paired t-test, and then all participants via independent samples t-test. The concentrations were either left as raw values, expressed as proportion relative to baseline (Prop.), or simple subtractive difference from baseline (Diff.). Degrees of freedom (df) vary due to problems obtaining particular blood draws and adjustments due to inequality of variance (Levene’s test). No correction for multiple comparisons has been applied to the associated *p* values, nevertheless none of these comparisons approaches significance.

|  |  | **df** | **P** |
| --- | --- | --- | --- |
| **Paired** | **Raw** | 29 | .316 |
|  | **Prop.** | 30 | .350 |
|  | **Diff.** | 28 | .235 |
| **Independent** | **Raw** | 58 | .744 |
|  | **Prop.** | 46 | .387 |
|  | **Diff.** | 56 | .315 |

**Table S5. Oxytocin t-test results comparing trust and mistrust.** Oxytocin concentrations were compared using both independent-samples and paired *t*-tests for task rounds in which the participant themselves (Self) demonstrated trust (by adhering to an agreed-upon strategy) vs. mistrust (not keeping with the agreed-upon strategy). Similarly, rounds in which the participants’ partner (Partner) demonstrated trustworthiness by adhering to the strategy vs. demonstrating untrustworthiness by not keeping with the agreed-upon strategy were also compared. These values were compared both before (Pre) and after (Post) each round. The concentrations were either left as raw values, expressed as proportion relative to baseline (Prop.), or simple subtractive difference from baseline (Diff.). Degrees of freedom (df) vary due to variance in participant behavior, problems obtaining particular blood draws, and adjustments due to inequality of variance (via Levene’s test). In the paired-samples tests, df are much lower due to the limited number of participants who exhibited or experienced both trust and mistrust in a session. Based on Zak, Kurzban, and Metzner (2005), the only *a* *priori* comparisons were the pre and post-round, raw values for self-behavior. Neither of these tests approached significance. The only test that is a possibly significant result is the paired test for pre-round difference from baseline based on partner behavior (*p*=.014, uncorrected). This result did not approach significance after applying either the conservative Bonferroni correction for multiple comparisons or the less-conservative False Discovery Rate procedure (Benjamini & Yekutieli, 2001, with q=.05).

|  |  |  | **Independent** | | **Paired** | |
| --- | --- | --- | --- | --- | --- | --- |
|  |  |  | **df** | **p** | **df** | **p** |
| **Pre** | **Self** | **Raw** | 76 | .262 | 14 | .217 |
|  |  | **Prop.** | 77 | .691 | 15 | .753 |
|  |  | **Diff.** | 76 | .478 | 14 | .793 |
|  | **Partner** | **Raw** | 82 | .681 | 20 | .014 |
|  |  | **Prop.** | 82 | .452 | 20 | .074 |
|  |  | **Diff.** | 82 | .147 | 20 | .137 |
| **Post** | **Self** | **Raw** | 76 | .437 | 14 | .344 |
|  |  | **Prop.** | 18.69 | .192 | 13 | .191 |
|  |  | **Diff.** | 17.11 | .303 | 14 | .193 |
|  | **Partner** | **Raw** | 82 | .756 | 20 | .399 |
|  |  | **Prop.** | 80 | .450 | 19 | .102 |
|  |  | **Diff.** | 80 | .603 | 20 | .388 |


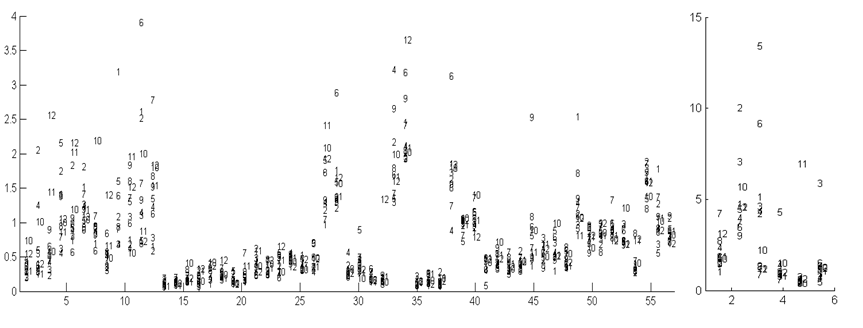


**Figure S1. All oxytocin concentrations, by participant and sample number, in Experiment Two.** Each number is the sample number in sequence for that subject, with different subjects (using arbitrary subject identifiers) on the x-axis. The y-axis is oxytocin concentration in pg/mL. Subjects with overall higher concentration values are shown on a secondary y-axis. Not shown is the one participant who produced three values at 39, 44, and 53 pg/mL.

**Figure S2. Time course of average oxytocin concentration**. Mean and standard errors for each sampling point in Experiment Two, calculated across all 64 participants. Lunch for all participants was provided between Draws 6 and 7; the total time span from draw 1 to 12 was approximately 6 hours. Consistent with previous results (e.g. Amico, Tenicela, Johnston, & Robinson, 1983), we did not observe significant diurnal variation in plasma oxytocin. We also did not observe any significant differences associated with consuming a meal.

**
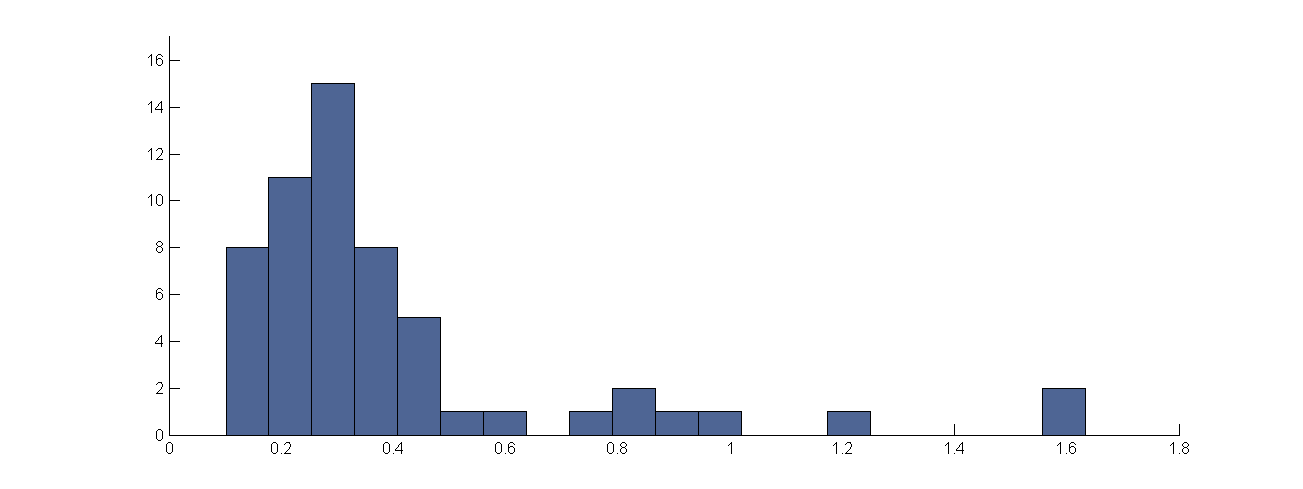
Figure S3. Coeffecients of variance in Experiment Two.** Coefficients of variance across participants in Experiment Two, calculated across the 12 samples rather than across the two replicates per sample. Standard deviations are less than the mean for all but three participants.


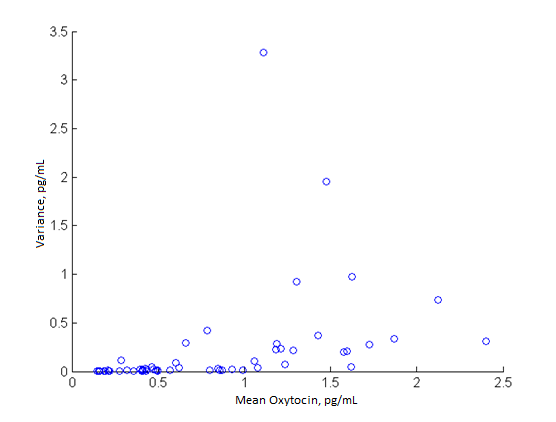


**Figure S4. Mean concentration vs. variance in Experiment Two.** Plot of mean oxytocin concentration, calculated within participants over the 12 samples, against the variance, excepting the two participants who produced values greater than 10 pg/mL.

**Text S3. Supplementary Refereces.**

Amico, J. A., Seif, S. M., & Robinson, A. G. (1981) Oxytocin in human plasma: correlation with neurophysin and stimulation estrogen. *Journal of Clinical Endocrinology & Metabolism*, *52*, 988-93

Amico, J. A., Tenicela, R., Johnston, J., & Robinson, A. G. (1983). A time-dependent peak of oxytocin exists in cerebrospinal fluid but not in plasma of humans. *Journal of Clinical Endocrinology & Metabolism*, *57*(5), 947-951.

Bagdy, G. G. and Arato, M. (1998) Gender-dependent dissociation between oxytocin but not ACTH,

cortisol or TSH responses to m-chlorophenylpiperazine in healthy subjects. Psychopharmacology, 136 (4), 342-348. doi:10.1007/s002130050576

Bello D, White-Traut R, Schwertz D, Pournajafi-Nazarloo H, Carter CS. (2008) An exploratory study of neurohormonal responses of healthy men to massage. J Altern Complement Med,14, 387-394.

Benjamini, Y. & Yekutieli, D. (2001). The control of the false discovery rate in multiple testing under dependency. *The Annals of Statistics*, 29(4), 1165-1188.

Billhult A, Lindholm C, Gunnarsson R, Stener-Victorin E (2008). The effect of massage on cellular immunity, endocrine and psychological factors in women with breast cancer-a randomized controlled clinical trial. Auton Neurosci, 140, 88-95.

Borg, J., Simren, M., & Ohlsson, B. (2011) Oxytocin reduces satiety scores without affecting the volume of nutrient intake or gastric emptying rate in healthy subjects. Neurogastroenterology and Motility, 23(1), 56-E5. doi: 10.1111/j.1365-2982.2010.01599.

Borg, J., Melander, O., Johansson, L., Uvnäs-Moberg, K., Rehfeld, J., & Ohlsson, B. (2009). Gastroparesis is associated with oxytocin deficiency, oesophageal dysmotility with hyperCCKemia, and autonomic neuropathy with hypergastrinemia. BMC Gastroenterology, 9(1), 17.

Carter, C. S., Pournajafi-Nuzarloo, H., Kramer, K. M., Ziegler, T. E., White-Traut, R., & Bello, D. (2007). Behavioral associations and potential as a salivary biomarker. Annals of the New York Academy of Sciences, 1098, 312-322.

Chicharro, J. L., Hoyos, J., Bandrés, F., Gómez Gallego, F., Pérez, M. , & Lucía, A. (2001) Plasma oxytocin during intense exercise in professional cyclists. Hormone Research, 55(3), 155-159. Accessible online at:

www.karger.com/journals/hre

Cyranowski JM, Hofkens TL, Frank E, Seltman H, Cai HM, Amico JA. (2008) Evidence of dysregulated peripheral oxytocin release among depressed women. Psychosom Med, 70, 967-975.

Domes G, Lischke A, Berger C, Grossmann A, Hauenstein K, Heinrichs M, Herpertz SC. (2010) Effects of intranasal oxytocin on emotional face processing in women. Psychoneuroendocrinology, 35, 83-93.

Dumont, G. H., Sweep, F. J., van der Steen, R. R., Hermsen, R. R., Donders, A. T., Touw, D. J., & ... Verkes, R. J. (2009) Increased oxytocin concentrations and prosocial feelings in humans after ecstasy (3,4-ethylenedioxymethamphetamine) administration. Social Neuroscience, 4(4), 359-366. doi:10.1080/17470910802649470

Feldman, R., Weller, A., Zagoory-Sharon, O., & Levine, A. (2007) Evidence for a neuroendocrinological foundation of human affiliation: Plasma oxytocin levels across pregnancy and the postpartum period predict mother-Infant bonding. Psychological Science, 18(11), 965-970. Doi: 10.1111/j.1467-9280.2007.02010.x

Feldman, R., Gordon, I., Schneiderman, I., Weisman, O., & Zagoory-Sharon, O. (2010). Natural variations in maternal and paternal care are associated with systematic changes in oxytocin following parent–infant contact. Psychoneuroendocrinology, 35(8), 1133-1141.

Floyd, K., Pauley, P. M., and Hesse, C. (2010) Communication Monographs, 77(4), 618-636. DOI: 10.1080/03637751.2010.498792

Goldman, M., Marlow-O’Connor, M., Torres, I., & Carter, C. S. (2008) Diminished plasma oxytocin in schizophrenic patients with neuroendocrine dysfunction and emotional deficits. Schizophrenia Research, 98, 247-255. Doi:101016/j.schres.2007.09.019

Gordon, I., Zagoory-Sharon, O., Leckman, J. F., & Feldman, R. (2010). Oxytocin and the development of parenting in humans. Biological psychiatry, 68(4), 377-382.

Gordon, I., Zagoory-Sharon, O., Schneiderman, I., Leckman, J.F., Weller, A, & Feldman, R. (2008) Oxytocin and cortisol in romantically unattached young

adults: Associations with bonding and psychological distress. Psychophysiology, 45, 349–352. DOI: 10.1111/j.1469-8986.2008.00649.x

Gouin, J., Carter, C. S., Pournajafi-Nazarloo, H., Glaser, R., Malarkey, W. B., Loving, T. J., Stowell, J., & Kiecolt-Glaser, J. K. (2010) Marital behavior, oxytocin, vasopressin, and wound healing. Psychoneuroendocrinology, 35(7), 1082–1090. doi:10.1016/j.psyneuen.2010.01.009.

Green L, Fein D, Modahl C, Feinstein C, Waterhouse L, Morris M. (2001) Oxytocin and autistic disorder: alterations in peptide forms. Biol Psychiatry, 50, 609-613.

Grewen KM, Girdler SS, Amico J, Light KC. (2005) Effects of partner support on resting oxytocin, cortisol, norepinephrine, and blood pressure before and after warm partner contact. Psychosom Med, 67, 531-538.

Grewen, K. M., Light, K. C., Mechlin, B., & Girdler, S. S. (2008, Jun) Ethnicity is associated with alterations in oxytocin relationships to pain sensitivity in women. Ethnicity & Health, 13(3), 219-241. Doi: 10.1080/13557850701837310

Grewen KM, Davenport RE, Light KC. (2010) An investigation of plasma and salivary oxytocin responses in breast- and formula-feeding mothers of infants. Psychophysiology, 47, 625-632.

Hoge, E. A., Pollack, M.H., Kaufman, R. E., Zak, P. J., & Simon, N. M. (2008) Oxytocin levels in social anxiety disorder. CNS Neuroscience & Therapeutics, 14 (3), 165-170. doi: 10.1111/j.1755-5949.2008.00051.x

Holt-Lunstad J, Birmingham WA, Light KC. (2008) Influence of a ‘‘warm touch’’support enhancement intervention among married couples on ambulatory blood pressure, oxytocin, alpha amylase, and cortisol. Psychosom Med, 70, 976-985.

Jansen, Lucres M. C.; Gispen-de Wied, Christine C.; Wiegant, Victor M.; Westenberg, Herman G. M.; Lahuis, Bertine E.; Engeland, Herman. Journal of Autism and Developmental Disorders vol. 36 issue 7 October 2006. p. 891 – 899

Keri, S., Kiss, I., & Keleman, O. (2009, Aug) Sharing secrets: Oxytocin and trust in schizophrenia. Social Neuroscience 4 (4), 287-293. Doi: 10.1080/17470910802319710

Lee R, Garcia F, Van de Kar LD, Hauger RD, Coccaro EF. (2003) Plasma oxytocin in response to pharmaco-challenge to D-fenfluramine and placebo in healthy men. Psychiatry Res, 118, 129-136.

Light, K. C., Smith, T. E., Johns, J. M., Brownley, K. A., Hofheimer, J. A., & Amico, J. A. (2000) Oxytocin responsitivity in mothers of infants: A preliminary study of relationships with blood pressure during laboratory stress and normal ambulatory activity. Health Psychology, 19(6), 560-567. doi:10.1037/0278-6133.19.6.560

Marazziti, D., Dell'Osso, B., Baroni, S., Mungai, F., Catena, M., Rucci, P., & ... Dell'Osso, L. (2006) A relationship between oxytocin and anxiety of romantic attachment. Clinical Practice And Epidemiology In Mental Health, 2:28. doi:10.1186/1745-0179-2-28

Miller, S. C., Kennedy, C., Devoe, D., Hickey, M., Nelson, T., & Kogan, L. (2009) An examination of changes in oxytocin levels in men and women before and after interaction with a bonded dog. Anthrozoos, 22(1), 31-42. Doi: 10.2752/175303708X390455

Modahl, C., Green, L., Fein, D., Morris, M., Waterhouse, L., Feinstein, C. & Levin, H. (1998) Plasma oxytocin levels in autistic children. Biological Psychiatry, 43(4), 270-277.

Nilsson, U. (2009, Aug ) Soothing music can increase oxytocin levels during bed rest after

open-heart surgery: a randomised control trial. Journal of Clinical Nursing, 18 (15), 2153-2161. doi: 10.1111/j.1365-2702.2008.02718.x

Ohlsson, B., Rehfeld, J. F., & Forsling, M. L. (2004) Oxytocin and cholecystokinin secretion in women with colectomy. BMC Gastroenterology, 425-426. doi:10.1186/1471-230X-4-25

Parker KJ, Kenna HA, Zeitzer JM, Keller J, Blasey CM, Amico JA, Schatzberg AF. (2010) Preliminary evidence that plasma oxytocin levels are elevated in major depression. Psychiatry Res, 178, 359-362.

Péqueux, C. C., Hendrick, J. C., Hagelstein, M. T., Geenen, V. V., & Legros, J. J. (2001) Novel plasma extraction procedure and development of a specific enzyme-immunoassay of oxytocin: application to clinical and biological investigations of small cell carcinoma of the lung. Scandinavian Journal Of Clinical & Laboratory Investigation, 61(5), 407-415. doi:10.1080/003655101316911468

Rahm, V., Hallgren, A., Högberg, H., Hurtig, I., & Odlind, V. (2002, Nov) Plasma oxytocin levels in women during labor with or without epidural analgesia: A prospective study. Acta Obstetricia et Gynecologica Scandinavica, 81(11), 1033-1039. Doi: 10.1034/j.1600-0412.2002.811107.x

Rubin, L. H., Carter, C. S., Drogos, L., Pournajafi-Nazarloo, H., Sweeney, J. A., & Maki, P. M. (2010) Peripheral oxytocin is associated with reduced symptom severity in schizophrenia. Schizophrenia Research, 124, 13-21. doi:10.1016/j.schres.2010.09.014

Salonia A, Nappi RE, Pontillo M, Daverio R, Smeraldi A, Briganti A, Fabbri F, Zanni G, Rigatti P, Montorsi F. (2005) Menstrual cycle-related changes in plasma oxytocin are relevant to normal sexual function in healthy women. Horm Behav 2005, 47, 164-169.

Szeto A, McCabe PM, Nation DA, Tabak BA, Rossetti MA, McCullough ME, Schneiderman N, Mendez AJ (2011) Evaluation of enzyme immunoassay and radioimmunoassay methods for the measurement of plasma oxytocin. Psychosom Med 73(5): 393-400.

Tabak BA, McCullough ME, Szeto A, Mendez AJ, McCabe PM. (2011) Oxytocin indexes relational distress following interpersonal harms in women. Psychoneuroendocrinology, 36, 115-122.

Taylor SE, Saphire-Bernstein S, Seeman TE. (2010) Are plasma oxytocin in women and plasma vasopressin in men biomarkers of distressed pair-bond relationships? Psychol Sci, 21, 3-7.

Tops, M., VanPeer, J. M., Korf, J., Wijers, A. A., & Tucker, D. M. (2007) Anxiety, cortisol, and attachment predict plasma oxytocin. Psychophysiology, 44(3), 444-449. doi:10.1111/j.1469-8986.2007.00510.x

Turner RA, Altemus M, Yip DN, Kupferman E, Fletcher D, Bostrom A,Lyons DM, Amico JA. (2002) Effects of emotion on oxytocin, prolactin, and ACTH in women. Stress, 5, 269-276.

Uckert S, Becker AJ, Ness BO, Stief CG, Scheller F, Knapp WH, Jonas U.(2003) Oxytocin plasma levels in the systemic and cavernous blood of healthy males during different penile conditions. World J Urol, 20, 323-326.

Van Londen, L., Goekoop, J. G., Zwinderman, A. H., Lanser, J. B. K., Wiegant, V. M., & De Wied, D. (1998). Neuropsychological performance and plasma cortisol, arginine vasopressin and oxytocin in patients with major depression. Psychological Medicine, 28(02), 275-284.

Weisman, O., Zagoory-Sharon, O., Schneiderman, I., Gordon, I., & Feldman, R. (2012). Plasma oxytocin distributions in a large cohort of women and men and their gender-specific associations with anxiety. Psychoneuroendocrinology.

Wikstrom, S., Gunnarsson, T., and Nordin, C. (2003) Tactile stimulus and neurohormonal response: A pilot study. Intern. J. Neuroscience, 113:787–793. DOI: 10.1080/00207450390200954

Wolff K, Tsapakis EM, Winstock AR, Hartley D, Holt D, Forsling ML, Aitchison KJ. (2006) Vasopressin and oxytocin secretion in response to the consumption of ecstasy in a clubbing population. J Psychopharmacol, 20, 400-410.

Zak, P. J., Kurzban, R., & Matzner, W. T. (2005) Oxytocin is associated with human trustworthiness. Hormones and Behavior, 48, 522-527. doi:10.1016/j.yhbeh.2005.07.009

Zhang, G., Zhang, Y., Fast, D. M., Lin, Z., & Steenwyk, R. (2011). Ultra sensitive quantitation of endogenous oxytocin in rat and human plasma using a two-dimensional liquid chromatography–tandem mass spectrometry assay. Analytical Biochemistry, 416(1), 45-52.
